# Serum levels of C1q/TNF-related protein-1 (CTRP-1) are closely associated with coronary artery disease

**DOI:** 10.1186/s12872-016-0266-7

**Published:** 2016-05-11

**Authors:** Huizhong Wang, Ru Wang, Dayong Du, Fangliang Li, Yuntian Li

**Affiliations:** Clinical Laboratory, The 305 Hospital of the People’s Liberation Army, Beijing, 100017 China; Department of Cardiology, The 305 Hospital of the People’s Liberation Army, Beijing, 100017 China

**Keywords:** Complement C1q tumor necrosis factor related protein 1, Coronary artery disease, Acute myocardial infarction

## Abstract

**Background:**

Complement C1q tumor necrosis factor related proteins (CTRPs) have been proved to have diverse biological influences on cardiovascular system. CTRP 1 is a member of the CTRP superfamily, however, the relevance with coronary artery disease (CAD) are seldom explored. This study was designed to investigate the correlation between serum levels of CTRP 1 and CAD.

**Methods:**

CTRP 1 levels of 150 CAD patients and 50 non-CAD subjects were determined by enzymelinked immunosorbent assay. Further analysis of CTRP 1 levels in different stages and lesion vessels of CAD were conducted.

**Results:**

Serum levels of CTRP 1 in CAD patients were significantly elevated, and it was increased with the severity of CAD. CTRP 1 level in acute myocardial infarction group was much higher than that in stable/unstable angina and non-CAD groups. And significant differences of CTRP 1 were also found between single-vessel disease and triple-vessel disease. Multiple logistic regression analysis showed that CTRP 1 was an independent risk factor of the occurrence of myocardial infarction.

**Conclusions:**

Increased serum CTRP 1 levels were closely associated with the prevalence and severity of CAD, it might be regarded as a marker for myocardial infarction.

## Background

Complement C1q tumor necrosis factor related proteins (CTRPs) superfamily is a cluster of adipokines, currently, 15 members are identified [1]. The highly conserved family of proteins share a similar modular organization to adiponectin and contain four distinct domains [2]. The members of CTRPs participate in the regulation of physiological and pathological processes, such as glucose and lipid metabolism, inflammation, myocardial protection, vasodilatation. CTRP 1 is identified as an adipokine, its expression is greatly increased in adipose tissue of *db/db* mice and Zucker diabetic fatty *(fa/fa)* rats [3]. It has been reported that the levels of CTRP 1 were significantly up-regulated in hypertensive patients’ serum [4]. Furthermore, CTRP 1 prevents collagen-induced platelet aggregation by specific blockade of von Willebrand’s factor binding to collagen, which suggests that CTRP 1 could be acted as a potent antithrombotic factor [5]. A recent study revealed that serum CTRP 1 levels were significantly increased in patients with type 2 diabetes mellitus, CTRP 1 was a novel adipokine associated with type 2 diabetes mellitus in humans [6]. The study by Shen and her colleagues [7] demonstrated that CTRP 1 could inhibit in vitro angiogenesis of endothelial progenitor cells from patients with severe coronary artery disease.

Coronary artery disease (CAD) is one of the most prevalent cardiovascular diseases, it is a group of diseases that included: stable angina, unstable angina, myocardial infarction, and sudden coronary death [8]. In 2013, CAD was the most common cause of death globally [9]. Multiple risk factors for CAD have been identified, including smoking, family history, hypertension, obesity, diabetes, lack of exercise, stress, and high blood lipids [10]. CTRP has also been shown to have diverse biological influences on cardiovascular system [7]. Pervious observations showed that CTRP 1 is associated with metabolic and vascular disorders, and those disorders are contributed to the pathogenesis of angiocardiopathy.

Among the extensive researches on the pathogenesis of cardiovascular disease, the role of adipokines has been gradually drawn more and more researchers’ attention [11]. Adipose tissue is not only considered as energy tissue, but also as an important endocrine organ that derives a number of biologically active adipokines. These adipokines exert the extraordinary biological functions. CTRP 1 is a member of the CTRP superfamily and express at high levels in adipose tissues. But there were little research explored the relevance of CTRP 1 with CAD. Hence, we developed a study regarding CTRP 1 and its relation with the prevalence or severity of CAD.

## Methods

### Subjects

A total of 200 participants who have underwent coronary angiography successfully at the Department of Cardiology, The 305 Hospital of the People’s Liberation Army were enrolled in the study between January 2010 and January 2012. The subjects included 133 males and 67 females, the average age was 61.11 ± 11.83 years. Stable and unstable angina pectoris were determined by American College of Cardiology (ACC)/American Heart Association (AHA) 2007 guidelines [12], the diagnosis of acute myocardial infarction was performed according to the clinical history, electrocardiogram and serum myocardial enzymogram. With the reference of their clinical diagnosis and coronary angiography results, they were divided into CAD group (*n* = 150) and non-CAD group (*n* = 50, with normal coronary artery angiogram). And CAD group further grouped into 3 subgroups by their clinical course: stable angina (SA) group (*n* = 48), unstable angina (UA) group (*n* = 43) and acute myocardial infarction (AMI) group (*n* = 59). The participants with cerebral infarction, thrombotic diseases, valvular heart disease, severe hepatic and renal dysfunction, tumors, infections and hematologic disorders were excluded. All of them were given written informed consent. This study was approved by the ethics committee of The 305 Hospital of the People’s Liberation Army.

### Methods

Blood samples were obtained within 24 h of admission from all the participants. Peripheral venous blood cells were isolated by density gradient centrifugation and the serum was stored at −70 °C. CTRP 1 levels were determined quantitatively with enzyme-linked immunosorbent assay using the commercially available ELISA kit (Cloud-Clone Corp) and followed the manufacturer’s recommendations. Other serum indexes were measured by routine methods using a fully automatic biochemical analyzer (UniCel DxC 800 Synchron, Beckman Coulter, Inc., USA).

The Seldinger technique was used in the coronary angiography. A GE300 cardiovascular angiographic system was used to show lesion location. According to different parts involved, the lesion can be classified into left main, anterior descending, circumflex and right coronary artery lesion. A stenosis ≥ 50 % was considered as CAD, and left main stenosis ≥ 50 % was considered as double-vessel disease.

### Statistical analysis

Statistical analyses were performed using SPSS 17.0 software. The measurement data were conducted by normality test. Continuous variables were presented as mean ± standard deviation (SD). The difference of measurement data was compared with *t* test between groups. Tukey’s test was used for pairwise comparison between groups. Count data were assessed using chi-square test. Logistic regression analysis was used to identify the association between CAD and other variables. All statistical tests used a *P* value of 0.05 in a two-tailed test as statistically significant.

## Results

### Clinical characteristics

Clinical characteristics of CAD patients and non-CAD subjects are listed in Table 1. The proportion of male, smoking and diabetes were observed more frequently in CAD group. CAD patients had significantly higher levels of glucose, creatinine (CRE) and CTRP 1 than non-CAD group. High density lipoprotein-cholesterol (HDL-c) level was lower in CAD group than that in non-CAD group. There were no statistical differences in triglyceride (TG), total cholesterol (TC), low density lipoprotein-cholesterol (LDL-c), urea nitrogen and uric acid between the two groups.

**Table 1:** Clinical characteristics of participants in CAD and non-CAD groups

	Non-CAD (*n* = 50)	CAD (*n* = 150)	*P*
Male (%)	24 (48)	109 (72.7)	0.001^**^
Age (years)	59.88 ± 9.31	61.57 ± 11.77	0.881
Smoking (%)	8 (16)	56 (37.3)	0.005^**^
Alcohol (%)	8 (16)	26 (17.3)	0.967
Hypertension (%)	18 (36)	77 (51.3)	0.06
Diabetes (%)	8 (16)	48 (32)	0.029^*^
Hyperlipidaemia (%)	16 (32)	60 (40)	0.313
TG (mmol/L)	1.60 ± 1.80	1.70 ± 1.41	0.732
TC (mmol/L)	4.13 ± 0.85	4.02 ± 1.17	0.506
HDL-C (mmol/L)	1.11 ± 0.20	0.90 ± 0.25	0.02^*^
LDL-C (mmol/L)	2.40 ± 0.85	2.37 ± 1.02	0.857
Glucose (mmol/L)	5.59 ± 1.06	6.53 ± 2.46	0.003^**^
Urea nitrogen (mmol/L)	4.94 ± 1.29	5.46 ± 1.59	0.102
CRE (μmol/L)	66.20 ± 14.92	75.48 ± 19.07	0.02^*^
Uric acid (μmol/L)	311.06 ± 77.45	338.86 ± 106.21	0.156
CTRP1 (ng/mL)	6.91 ± 2.80	10.20 ± 8.89	0.031^*^

Compared with non-CAD group, ^*^ *P* < 0.05, ^**^ *P* < 0.01

### Relationship between CTRP 1 and different stages of CAD

**Fig. 1:**
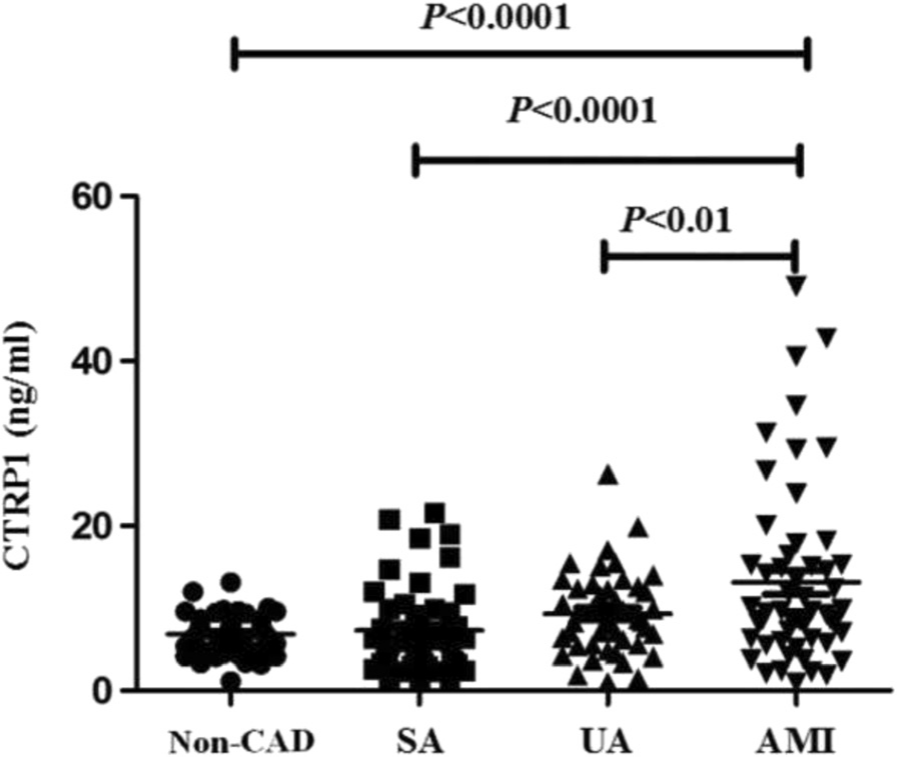
CTRP 1 levels in patients with SA, UA, AMI and non-CAD subjects

**Fig. 2:**
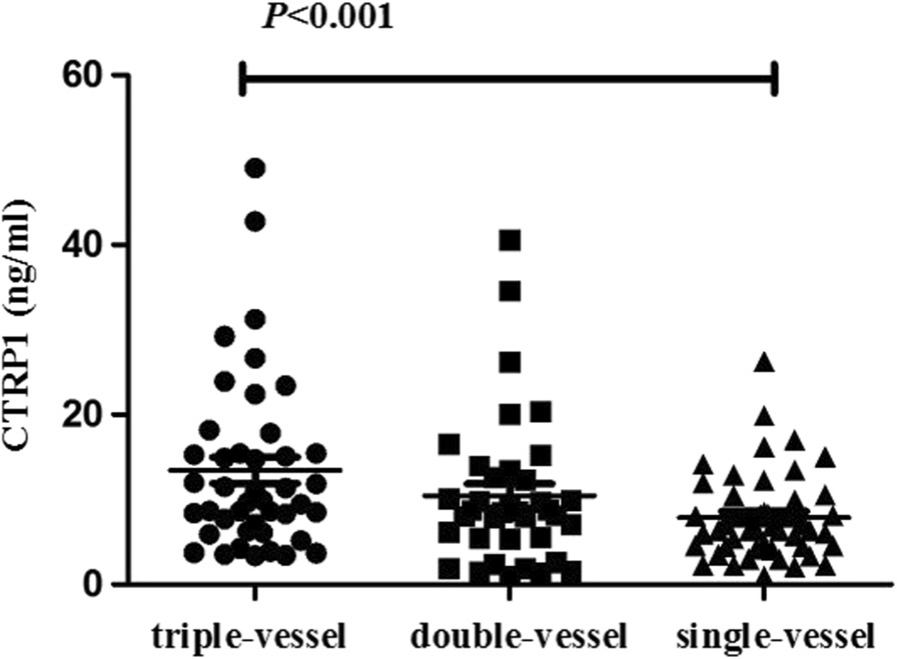
CTRP 1 levels in patients with single-vessel, double-vessel and triple-vessel disease

To further clarify the correlation between the increased levels of CTPR 1 and the severity of CAD, we compared CTRP 1 level of different subgroups. CAD patients were grouped by the development of the disease, the CTRP 1 level (Fig. 1) in SA, UA and AMI group was 7.33 ± 6.46, 9.30 ± 4.94 and 12.52 ± 10.60 ng/mL, respectively. CTRP 1 levels in AMI group was significantly higher than that in SA, UA and non-CAD groups (*P* < 0.01). But the differences were not significant between SA, UA and non-CAD groups. When grouped according to different coronary lesion vessels, the CTRP 1 level (Fig. 2) in single-vessel, double-vessel and triple-vessel disease group was 7.82 ± 5.14, 10.40 ± 8.66 and 13.42 ± 10.11 ng/mL, respectively. CTRP 1 levels increased with the coronary lesion vessels, it was markedly different between single-vessel and triple-vessel disease group (*P* < 0.01). There had no difference in single-vessel and double-vessel disease group, double-vessel and triple-vessel disease group.

### Association of CTRP 1 levels with myocardial infarction

To confirm the association between CTRP 1 and myocardial infarction, multiple logistic regression analysis was performed between AMI and non-CAD groups (Table 2). Gender, age, systolic/diastolic blood pressure, glucose, TG, TC, HDL-c, LDL-c and CTRP 1 were regarded as independent variables. The result demonstrated that gender, glucose and CTRP 1 were independent risk factors associated with myocardial infarction.

**Table 2:** Logistic regression analysis of related risk factors for CAD

	OR	95 % CI	*P*
Male	0.220	0.052–0.920	0.018
Systolic pressure	1.017	0.954–1.084	0.614
Diastolic pressure	0.957	0.864–1.061	0.406
Glucose (mmol/L)	1.714	1.022–2.875	0.041
TG (mmol/L)	0.787	0.321–1.925	0.599
TC (mmol/L)	0.502	0.018–13.993	0.685
HDL-c (mmol/L)	0.562	0.010–30.630	0.777
LDL-c (mmol/L)	1.734	0.072–41.723	0.735
CTRP1 (ng/mL)	1.319	1.053–1.652	0.016

## Discussion

Our study demonstrated that the CTRP 1 levels were increased in patients with CAD compared to non-CAD subjects, and the CTRP 1 levels were on an upward trend with the progress of disease. Multiple logistic regression analysis showed that increased CTRP 1 level was independent risk factor of myocardial infarction.

Some studies have indicated that CTRP family members, including adiponectin, CTRP 3 and CTRP 9, were involved in the regulation of cardiovascular disease in mice and human. For example, CTRP 3 has been found to be associated with cardiac risk factors in human [13]. Yi [14] first demonstrated that the expression and production of CTRP 3 were significantly reduced after myocardial infarction, the administration of CTRP 3 could improve survival rate and restore cardiac function in mice with coronary artery occlusion. Animal experiments showed that CTRP 9 administration to mice significantly reduced infarct size and improved cardiac function [15], and CTRP 9 protected against acute cardiac injury following ischemia-reperfusion via an AMPK-dependent mechanism [16]. Furthermore, CTRP 9 was also a vasorelaxative adipocytokine that exerted vasculoprotective effects [17].

In recent study, increased serum CTRP 1 level in SA patients were observed [7]. Consistent with these findings, our data in this study indicated that CTRP 1 levels in CAD group were significantly higher than non-CAD group. It has also been reported that CTRP 1 levels were elevated with the disease progresses and coronary lesion vessels. All these observations proved that CTRP 1 level were closely related to the pathogenesis and severity of CAD. Previous studies have reported that CTPR 1 were significantly increased in subjects with hypertension [4], diabetes [6], as well as metabolic syndrome [18]. Because these diseases were the recognized risk factors for atherosclerosis, in order to test whether our findings were influenced by those factors, multivariate stepwise regression analysis was performed to evaluate the independent factors of myocardial infarction with the above factors. Gender, glucose and CTRP 1 were the independent risk factors. It was obvious that metabolic abnormalities induced by CAD affected CTRP 1 levels more or less, but the increased CTRP 1 levels probably not related to them completely. The body’s inflammatory states and mutual adjustment between other members in CTRP superfamily might also contribute the enhancement of CTRP 1.

## Conclusion

The actual role of CTRP 1 in the cardiovascular system is completely unknown [19]. However, the increased CTRP 1 were closed related with CAD, suggesting CTRP1 levels might serve as a predictor of CAD. Therefore, CTRP 1 levels in CAD patients should be paid highly attentions. There were important potential for CTRP 1 to be used as a novel biomarker to evaluate cardiovascular risk, but the assumption needed future clarification after acquiring the exact mechanism. The future study should conduct on a larger population, and potential confounding factors such as obesity should be excluded. Meanwhile, as a member of complex CTRP family, the interrelationship of other members with different biological function should be considered.

### Ethics approval and consent to participate

This study was approved by the ethics committee of The 305 Hospital of the People’s Liberation Army. All of participants were given written informed consent.

### Availability of data and materials statement

The datasets supporting the conclusions of this article are included within the article.

## Abbreviations

AMI: acute myocardial infarction
CAD: coronary artery disease
CRE: creatinine
CTRPs: complement C1q tumor necrosis factor related proteins
HDL-c: high density lipoprotein-cholesterol
LDL-c: low density lipoprotein-cholesterol
SA: stable angina
TC: total cholesterol
TG: triglyceride
UA: unstable angina
